# Development and Optimization of a Novel Soft Sensor Modeling Method for Fermentation Process of *Pichia pastoris*

**DOI:** 10.3390/s23136014

**Published:** 2023-06-29

**Authors:** Bo Wang, Jun Liu, Ameng Yu, Haibo Wang

**Affiliations:** Key Laboratory of Agricultural Measurement and Control Technology and Equipment for Mechanical Industrial Facilities, School of Electrical and Information Engineering, Jiangsu University, Zhenjiang 212013, China; wangbo@ujs.edu.cn (B.W.); 2222107008@stmail.ujs.edu.cn (A.Y.); 2212107036@stmail.ujs.edu.cn (H.W.)

**Keywords:** soft sensor, improved particle swarm algorithm, least squares support vector machine, transfer learning, *Pichia pastoris*

## Abstract

This paper introduces a novel soft sensor modeling method based on BDA-IPSO-LSSVM designed to address the issue of model failure caused by varying fermentation data distributions resulting from different operating conditions during the fermentation of different batches of *Pichia pastoris*. First, the problem of significant differences in data distribution among different batches of the fermentation process is addressed by adopting the balanced distribution adaptation (BDA) method from transfer learning. This method reduces the data distribution differences among batches of the fermentation process, while the fuzzy set concept is employed to improve the BDA method by transforming the classification problem into a regression prediction problem for the fermentation process. Second, the soft sensor model for the fermentation process is developed using the least squares support vector machine (LSSVM). The model parameters are optimized by an improved particle swarm optimization (IPSO) algorithm based on individual differences. Finally, the data obtained from the *Pichia pastoris* fermentation experiment are used for simulation, and the developed soft sensor model is applied to predict the cell concentration and product concentration during the fermentation process of *Pichia pastoris*. Simulation results demonstrate that the IPSO algorithm has good convergence performance and optimization performance compared with other algorithms. The improved BDA algorithm can make the soft sensor model adapt to different operating conditions, and the proposed soft sensor method outperforms existing methods, exhibiting higher prediction accuracy and the ability to accurately predict the fermentation process of *Pichia pastoris* under different operating conditions.

## 1. Introduction

The *Pichia pastoris* expression system is a eukaryotic expression system that has developed in the past decade and is one of the most successful foreign protein expression systems [1]. Compared with other expression systems, *Pichia pastoris* has significant advantages in processing, secretion, post-translational modifications, and glycosylation of expressed products, and has been widely applied [2]. Over 1,000 proteins have been efficiently expressed using the *Pichia pastoris* expression system. High-density fermentation is an important strategy for improving foreign protein expression levels in *Pichia pastoris* [3]. To effectively increase the expression level of secreted foreign proteins in *Pichia pastoris*, the fermentation process needs to be dynamically regulated and optimized in real-time by changing the fermentation environment and cultivation conditions to find the optimal environmental parameters for improving the secretion effect of foreign proteins [4]. However, *Pichia pastoris* fermentation is a complex, nonlinear, and uncertain process with multiple variables and time-varying properties [5,6]. Due to the actual process technology and cost reasons, key biochemical parameters that directly reflect fermentation process quality, such as cell concentration and product concentration, cannot be directly measured online and can only be estimated roughly through offline sampling and analysis [7]. This not only causes lagging information acquisition but also affects the correct judgment and decision-making of operators on real-time reaction status, while also limiting the implementation of optimization and control strategies. Therefore, there is an urgent need to find a method to achieve optimal estimation and prediction of key biochemical parameters during *Pichia pastoris* fermentation processes.

The soft sensor method is an effective solution to the problem of difficult online measurement of key biochemical parameters in biological fermentation processes. Many scholars worldwide have conducted in-depth research on soft sensor technology and achieved a series of results. Shao et al. [8] proposed a semisupervised Gaussian regression for the ammonia synthesis process, which achieved accurate real-time prediction of ammonia production concentration with fewer labeled samples. However, the accuracy parameter in Bayesian regularization needs to be manually predefined, which greatly reduces the accuracy of the model. Yuan et al. [9] used a supervised long short-term memory network to achieve soft sensor modeling of the penicillin fermentation process, which fully utilized the quality variables in the long short-term memory network and realized nonlinear dynamic modeling of the penicillin fermentation process with a good prediction effect. The limitation of this method is that the amount of computation and training time of the model are greatly increased due to adding the quality variable to each LSTM cell. Zheng et al. [10] proposed a real-time semisupervised extreme learning machine, which integrated semisupervised learning and just-in-time learning strategies into the modeling framework to establish a local prediction model, and fully utilized a large amount of unlabeled data information to achieve fast and accurate measurement of Mooney viscosity in the rubber mixing process. Chang et al. [11] proposed a consistent contrastive network to realize the time awareness and robustness of the soft sensor model, which overcame the limitations of manifold regularization and fully utilized abnormal data and unlabeled data information. The effectiveness of the consistent contrastive network was verified in the soft sensor modeling of ammonia and sulfur removal industrial processes. Fan et al. [12] proposed a soft sensor regression model based on the long short-term memory recurrent neural network in deep learning using the data obtained by the sensor, by designing the relative error loss and the normalized L1 loss function using the time step of the sensor to predict the measured value, to realize the detection of the wafer manufacturing process to reduce the recall rate of the wafer. The experimental results show that the proposed soft sensor model can realize various types of inspection and prediction in complex manufacturing processes. However, the generalization ability of this model is poor, and it can only be used in the manufacturing process of one working condition. Zhang et al. [13] deeply analyzed the relevant factors affecting the formation of glutamate, and proposed a soft sensor model based on fuzzy reasoning based on a support vector machine using the soft sensor method, and used the particle swarm optimization algorithm to optimize the key parameters to realize the control of glutamate concentration. The precise prediction of the model is optimized by using the fuzzy reasoning mechanism and the fuzzy basis function to optimize the kernel function of the support vector machine, which improves the anti-interference ability and adaptability of the model, whose prediction ability is good. However, the generalization ability of the model is insufficient, and the calculation process is relatively complicated, increasing the time cost. Han et al. [14], inspired by the adversarial network, used the adversarial domain adaptation method to improve the performance of the deep migration learning model and realize the accurate diagnosis of mechanical failures with small data volumes in industrial processes. However, this method must have sufficient target domain data. After the migration is completed, the target domain data in the actual industrial process are extremely limited, which affects the predictive performance of the model. Although the soft sensor models established in the above literature achieve accurate online prediction of key biological parameters, they do not consider the problem of model failure and performance degradation caused by the mismatch between modeling data and real-time data under different operating conditions in biological fermentation processes.

Regarding the abovementioned issue, [15] utilized deep transfer learning strategies to reduce the differences in data distribution between the source and target domains, effectively solving the problems of missing data and deterioration of soft sensor model performance in complex industrial processes. However, this method is only suitable for transferring from one working condition to another, which will reduce the ability of the model to fit. Ref. [16] proposed an online transfer learning technique based on slow feature analysis and variational Bayesian inference to improve the predictive performance of the target process, solving the problem of online measurement of water content in crude oil emulsions using steam-assisted gravity drainage technology and greatly improving production efficiency. Two weighting functions related to the transformation and emission equations are introduced and dynamically updated to quantify the transferability from the source domain to the target domain at each moment. Ref. [17] aims at the problem of online detection of key variables in industrial processes, proposes a soft sensor model based on variational mode decomposition, stacked enhanced autoencoder and transfer learning to achieve high-precision regression prediction, and introduces a transfer learning algorithm based on the maximum mean deviation in transfer learning to solve the domain under different operating conditions of the adaptation problem. However, the hyperparameters of the built model need to be manually selected, which greatly increases the prediction error of the model. Ref. [18] implements transfer learning by fine-tuning the weights of the network, freezing inner layers and updating outer layers. The method of transfer learning proposed in the literature realizes the prediction of complex industrial processes with small amounts of data. However, the prediction accuracy of this method is poor, and multiple parameters need to be manually set. Therefore, the application of transfer learning can effectively solve the dilemma of model failure and performance deterioration under different operating conditions. In summary, this paper proposes a multioperating condition transfer learning-based soft sensor modeling method for *Pichia pastoris* fermentation. First, to address the issue of significant data distribution differences between batches in the fermentation process, the BDA method in transfer learning is adopted to reduce the differences [19], and the fuzzy set concept is introduced to improve the BDA method, effectively converting the classification problem into a regression prediction problem of the fermentation process. Then, considering the nonlinearity and small sample characteristics of the *Pichia pastoris* fermentation process, the LSSVM is used as the basic model of the soft sensor process, and the adapted data are used to train the LSSVM model. The model is then optimized using an IPSO based on psychological mechanisms. Finally, the adapted target domain data are used to predict the *Pichia pastoris* cell concentration and product concentration through the constructed soft sensor model. The experimental results demonstrate that this soft sensor method is significantly superior to existing methods, has high prediction accuracy, and can accurately predict the *Pichia pastoris* fermentation process under different operating conditions. This paper makes significant contributions to current research on soft sensors. First, an IPSO algorithm is proposed to optimize the parameters of LSSVM, which greatly improves prediction accuracy. Compared with GWO and ABC optimization algorithms, the proposed IPSO algorithm has good dynamic performance and optimization performance. Second, this paper proposes to use the improved BDA algorithm in transfer learning to adapt the source domain and target domain to reduce the differences between different domains, so that the soft sensor model achieves good performance under multiworking conditions.

## 2. Methods

### 2.1. Principle and Solution of Balanced Distribution Adaptation

During the fermentation process of *Pichia pastoris*, the real-time data and modeling data distributions between different fermentation batches do not match due to varying operating conditions [20]. Soft sensor models established based on historical operational data may not be applicable to new batches, leading to model degradation and misalignment issues. Considering that transfer learning can learn useful information from other fermentation batches to assist in completing tasks for the target fermentation batch and does not require training and prediction data to conform to the requirement of independent and identically distributed data [21], it is an effective way to solve the problem of soft sensor modeling for the fermentation process with multiple operating conditions across different batches. Transfer learning has been widely used in medical images, industrial processes, and so on [22,23]. Transfer learning aims to improve the performance of target learners on target domains by transferring knowledge contained in different but related source domains [24]. Therefore, this article introduces a transfer learning strategy to construct a soft sensor model for the fermentation process of *Pichia pastoris*.

Data distribution adaptation is one of the most commonly used feature-based transfer learning methods [25]. The main idea behind this method is to use some transformations to bring the distance between data distributions of different domains closer together [26]. Currently, the most commonly used algorithm for this purpose is BDA, which reduces the joint probability distribution distance between the source and target domains to achieve transfer learning and improve the applicability and predictive accuracy of traditional soft sensor models. However, since the traditional BDA method is only suitable for solving classification problems [27], and soft sensor modeling for the fermentation process of *Pichia pastoris* is a regression problem, this article introduces the concept of fuzzy sets [28] into the BDA method to transform the classification problem into a regression problem and to achieve soft sensor modeling for the fermentation process of *Pichia pastoris*.

For a given labeled source domain data $Q_{s}={\left\{x_{s_{i}},y_{s_{i}}\right\}}_{i=1}^{n}$ and unlabeled target domain data $Q_{t}={\left\{x_{t_{j}},y_{t_{j}}\right\}}_{j=1}^{m}$ of the *Pichia pastoris* fermentation process, assuming the feature spaces are $\chi_{s}$ and $\chi_{t}$, respectively, and $\chi_{s}=\chi_{t}$, the label spaces are $Y_{s}$ and $Y_{t}$, respectively, and $Y_{s}=Y_{t}$, the marginal distributions are $P_{s}(x_{s})$ and $P_{t}(x_{t})$, respectively, and $P_{s}(x_{s})\neq P_{t}(x_{t})$, and the conditional distributions are $P_{s}(y_{s}|x_{s})$ and $P_{s}(y_{t}|x_{t})$, respectively, and $P_{s}(y_{s}|x_{s})\neq P_{s}(y_{t}|x_{t})$. The goal of BDA is to complete transfer learning by minimizing the marginal and conditional distributions between the source and target domains, which is minimizing the following equation.

(1) $$DIS(Q_{s},Q_{t})\approx (1-\mu )DIS(P(x_{s}),P(x_{t}))+\mu DIS(P(y_{s}|x_{s}),P(y_{t}|x_{t}))$$

where μ is the balance factor to adjust the distance between the two distributions, μ∈[0,1]. When μ→1, it indicates that data sets are similar, and the conditional distribution has a greater proportion. When μ→0, it indicates that the data sets are dissimilar, and the marginal distribution has a greater proportion.

Since there are no labels in the target domain $Q_{t}$, it is impossible to calculate the conditional probability distribution. Therefore, further training of a preclassifier is necessary to obtain soft labels $y_{t_{j}}$.

Let $Q_{s}^{c}=\{x_{i}|x_{i}\in Q_{s}\wedge y_{i}=c\}$ be the sample set of class *c* in the source domain *s*, and $Q_{t}^{c}=\{x_{i}|x_{i}\in Q_{t}\wedge y_{i}=c\}$ be the sample set of class *c* in the target domain *t*.

Using Maximum Mean Discrepancy (MMD) [29] to measure the distance between two neighboring domains, Equation (1) can be expressed as:

(2) $$DIS(Q_{s},Q_{t})\approx (1-\mu )|\;|\frac{1}{n}\sum_{i=1}^{n}x_{s_{i}}-\frac{1}{m}\sum_{j=1}^{m}x_{t_{j}}|\;{|}_{H}^{2}+\mu \sum_{c=1}^{C}|\;|\frac{1}{n_{c}}\sum_{x_{s_{i}}\in Q_{s}{}^{c}}x_{s_{i}}-\frac{1}{m_{c}}\sum_{x_{t_{j}}\in Q_{t}{}^{c}}x_{t_{j}}|\;{|}_{H}^{2}$$

where H is the Reproducing Kernel Hilbert Space [30], *c* denotes different class labels, and c∈{1,2,⋯,C}, *n* and *m* represent the number of samples in the source and target domains, respectively. $Q_{s}{}^{c}$ and $Q_{t}{}^{c}$ refer to class *c* samples in the source and target domains, respectively. $n_{c}$ and $m_{c}$, respectively, represent the number of samples in $Q_{s}{}^{c}$ and $Q_{t}{}^{c}$. The first term in Equation (2) computes the distance between the marginal probability distributions of the source and target domains, and the second term calculates the distance between the conditional probability distributions.

Considering that the soft sensor problem of *Pichia pastoris* fermentation process studied in this paper is a regression problem, while the BDA method is only applicable to classification problems, this paper introduces the fuzzy set method to improve the BDA method and make it applicable to regression problems.

**Figure 1:**
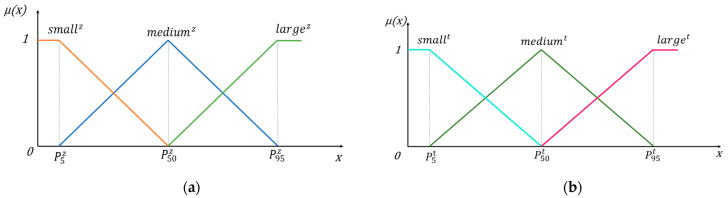
Percentage based fuzzy set division (**a**) Fuzzy sets in the source domain; (**b**) Fuzzy sets in the target domain.

In traditional classification problems, a sample can only belong to one class, while using the fuzzy set method allows a sample to belong to multiple classes to varying degrees, therefore, for the output ${\left\{y_{i}^{z}\right\}}_{i=1,\cdots ,n}$ of the *z*-th source domain, the 5th, 50th, and 95th percentile values are found and denoted as $p_{5}^{z},p_{50}^{z},p_{95}^{z}$. Three fuzzy sets denoted as ${\mathrm{small}}^{z}{,\;\mathrm{medium}}^{z},\;$${\mathrm{large}}^{z}$ are defined based on these percentiles, as shown in Figure 1.

Let the membership degree of $y_{i}^{z}$ in class *p* in the source domain be denoted as $\alpha_{ip}^{z}$, and the membership degree of $y_{i}^{t}$ in class *q* in the target domain be denoted as $\alpha_{iq}^{t}$, and normalize $\alpha_{ip}^{z}$ and $\alpha_{iq}^{t}$, as shown in Equations (3) and (4):

(3) $${\bar{\alpha }}_{ip}^{z}=\frac{\alpha_{ip}^{z}}{\sum_{i=1}^{n}\alpha_{ip}^{z}}\;p=1,2,3;i=1,2,\cdots ,n$$

(4) $${\bar{\alpha }}_{iq}^{t}=\frac{\alpha_{iq}^{t}}{\sum_{i=1}^{n}\alpha_{iq}^{t}}\;q=1,2,3;i=1,2,\cdots ,n$$

Based on Equations (3) and (4), Equation (2) can be represented as:

(5) $$DIS(Q_{s},Q_{t})\approx (1-\mu )|\;|\frac{1}{n}\sum_{i=1}^{n}x_{s_{i}}-\frac{1}{m}\sum_{j=1}^{m}x_{t_{j}}|\;{|}_{H}^{2}+\mu \sum_{c=1}^{3}|\;|\sum_{x_{s_{i}}\in D_{s}{}^{\left(c\right)}}{\bar{\alpha }}_{ic}^{z}x_{s_{i}}-\sum_{x_{t_{j}}\in D_{t}{}^{\left(c\right)}}{\bar{\alpha }}_{ic}^{t}x_{t_{j}}|\;{|}_{H}^{2}$$

Using matrix techniques, Equation (5) can be written in the following form:

(6) $$\begin{array}{l} DIS(Q_{s},Q_{t})\approx A^{T}X(1-\mu )M_{0}X^{T}A+A^{T}X\mu \sum_{c=1}^{3}M_{c}X^{T}A\\ =A^{T}X[(1-\mu )M_{0}+\mu M_{R}]X^{T}A \end{array}$$

where $M_{R}=M_{1}+M_{2}+M_{3}$, $M_{1},M_{2}$ and $M_{3}$ are matrices for maximum mean discrepancy, defined as follows:

(7) $${\left(M_{c}\right)}_{ij}=\left\{\begin{array}{ll} {\bar{\alpha }}_{ic}^{z}{\bar{\alpha }}_{jc}^{z} & x_{i},x_{j}\in Q_{s}^{c}\\ {\bar{\alpha }}_{ic}^{t}{\bar{\alpha }}_{jc}^{t} & x_{i},x_{j}\in Q_{t}^{c}\;\\ -{\bar{\alpha }}_{ic}^{z}{\bar{\alpha }}_{jc}^{z} & x_{i}\in Q_{s}^{c},x_{j}\in Q_{t}^{c}\\ -{\bar{\alpha }}_{ic}^{t}{\bar{\alpha }}_{jc}^{t} & x_{i}\in Q_{s}^{c},x_{j}\in Q_{t}^{c}\\ 0 & other \end{array}\right.$$

The calculation formula of $M_{0}$ is as follows:

(8) $${\left(M_{0}\right)}_{ij}=\left\{\begin{array}{ll} \frac{1}{n^{2}} & x_{i},x_{j}\in Q_{s}\\ \frac{1}{m^{2}} & x_{i},x_{j}\in Q_{t}\\ \frac{-1}{mn} & other \end{array}\right.$$

The objective function Equation (2) can be represented as:

(9) $$min\;tr(A^{T}X[(1-\mu )M_{0}+\mu M_{R}]X^{T}A)+\lambda |\;|A|\;{|}_{F}^{2}\;s.t.\;A^{T}XHX^{T}A=I,0\leq \mu \leq 1$$

where λ is the regularization parameter, *A* is the transformation matrix, *X* is the input matrix composed of $x_{s_{i}}$ and $x_{t_{j}}$, $||•{\left|\right|}_{F}^{2}$ is the Hilbert-Schmidt norm, *I* is the identity matrix, $I\in {\mathbb{R}}^{\left(n+m)*(n+m\right)}$, and *H* = *I* − (1/*n*)*E*, *E* is the identity matrix. By using the Lagrange multiplier method, the Lagrange function for Equation (9) is:

(10) $$L=tr(A^{T}X[(1-\mu )M_{0}+\mu M_{R}]X^{T}A)+\lambda |\;|A|\;{|}_{F}^{2}+tr([I-A^{T}XHX^{T}A]\phi )$$

where the Lagrange multiplier $\phi =(\phi_{1},\phi_{2},\cdots ,\phi_{d})$, set derivative ∂L/∂A=0, then the optimization problem can be transformed into a generalized eigenvalue decomposition problem, which can be expressed as:

(11) $$(X[(1-\mu )M_{0}+\mu M_{R}]X^{T}+\lambda I)A=XHX^{T}A\phi$$

The optimal transformation matrix *A* can be obtained by solving Equation (11).

By using the optimal transformation matrix *A*, the distributions of the source domain data and the target domain data can be adapted, thereby improving the applicability and prediction accuracy of the soft sensor model.

### 2.2. Improving Particle Swarm—Least Squares Support Vector Machine Algorithm

#### 2.2.1. Least Squares Support Vector Machine

The key biochemical parameters reflecting the real-time fermentation status and fermentation quality of *Pichia pastoris* (such as cell concentration and product concentration) are currently mainly obtained through offline sampling and laboratory analysis methods. However, this method is cumbersome and leads to long intervals between data collection for the same batch of fermentation, resulting in a limited number of actual collected data samples. In addition, the fermentation process has strong nonlinear characteristics. In light of the favorable performance of LSSVM in solving small sample, nonlinear, and high-dimensional regression tasks [31] and its fast solution speed and robust fitting ability, this paper applied LSSVM to soft sensor modeling of *Pichia pastoris* fermentation.

Suykens et al. [32] proposed the least LSSVM as a variant of the support vector machine (SVM). This method greatly reduces the complexity of the algorithm by using the sum of squared errors as the loss function. Given a dataset ${\left\{x_{i},y_{i}\right\}}_{i=1}^{l}$ with the input data $x_{i}\in R^{n}$ and the output data $y_{i}\in R^{n}$, the optimization objective of LSSVM can be represented as follows:

(12) $$\underset{\omega ,e}{\min\;}J(\omega ,e)=\frac{1}{2}\omega^{T}\omega +\frac{\gamma }{2}\sum_{i=1}^{l}e_{i}^{2}$$

Subject to:

(13) $$y_{i}[\omega^{T}\varphi (x_{i})+b]=1-e_{i}\;i=1,2,\cdots ,l$$

where ω represents the weight vector, φ(i) is a nonlinear function that maps the data to a high-dimensional space, γ is the regularization parameter, $e_{i}$ is the error introduced by the samples, and *b* is the constant bias. The optimization objective can be converted into a dual variable optimization problem using the Lagrange duality, which can be expressed as follows:

(14) $$\begin{array}{l} L(\omega ,b,e,\alpha )=J(\omega ,e)-\sum_{i=1}^{l}\alpha_{i}(\omega^{T}\varphi (x_{i})+b+e_{i}-y_{i})\\ =\frac{1}{2}\omega^{T}\omega +\frac{\gamma }{2}\sum_{i=1}^{l}e_{i}^{2}-\sum_{i=1}^{l}\alpha_{i}\{[\omega^{T}\varphi (x_{i})+b]+e_{i}-y_{i}\} \end{array}$$

where $\alpha_{i}$ is the Lagrange multiplier for the *i*-th constraint, according to the Karush–Kuhn–Tucker (KKT) conditions:

(15) $$\begin{array}{l} \frac{\partial L}{\partial \omega }=0\to \sum_{i=1}^{l}\alpha_{i}\varphi (x_{i})=\omega \\ \frac{\partial L}{\partial b}=0\to -\sum_{i=1}^{l}\alpha_{i}y_{i}=0\\ \frac{\partial L}{\partial e_{i}}=0\to \alpha_{i}=\gamma e_{i}\\ \frac{\partial L}{\partial \alpha_{i}}=0\to \omega^{T}\varphi (x_{i})+b+e_{i}-y_{i}=0 \end{array}$$

After eliminating ω,e, Equation (16) is obtained as follows:

(16) $$\left[\begin{array}{cc} 0 & E^{T}\\ E & \Omega +\gamma^{-1}I_{l} \end{array}\right]\left[\begin{array}{c} b\\ \alpha \end{array}\right]=\left[\begin{array}{c} 0\\ y \end{array}\right]$$

where $E={\left[1,\ldots ,1\right]}^{T}$, $I_{l}$ is an l×l identity matrix, $\alpha ={\left[\alpha_{1},\ldots ,\alpha_{l}\right]}^{T}$, and Ω is the kernel matrix, $\Omega \in {\mathbb{R}}^{N\times N}$, and $\Omega_{ij}=\varphi {\left(x_{i}\right)}^{T}\varphi (x_{j})=K(x_{i},x_{j})$ for a given RBF kernel function:

(17) $$K(x_{i},x_{j})=\exp(\frac{-{\left\|\left(x_{i}-x_{j}\right)\right\|}^{2}}{2\sigma^{2}})$$

Thus, the objective function can be derived as follows:

(18) $$f(x)=\sum_{i=1}^{l}\alpha_{i}K(x,x_{i})+b$$

where α,b are the solutions to Equation (16).

The predictive performance of LSSVM mainly depends on the regularization parameter γ and the kernel width σ, where γ balances the trade-off between fitting accuracy and model generalization ability, and σ affects the complexity of the distribution of the sample data in the mapped space. Traditional methods for parameter selection are often based on experience and trial, which may not guarantee regression accuracy and computational efficiency. To improve the LSSVM model, the IPSO algorithm is used to optimize the parameters (σ,γ) of LSSVM.

#### 2.2.2. Improved Particle Swarm Optimization

Particle swarm optimization (PSO) is an evolutionary algorithm used to solve global optimization problems [33]. However, PSO has certain limitations such as a lack of dynamic adaptability, which can result in local optima trapping and slow convergence speed [34]. Researchers have proposed improved PSO algorithms to address these limitations, including dynamic PSO and adaptive PSO [35]. These algorithms have shown promising results in enhancing PSO’s performance.

Yang Ge et al. [36] introduced a psychological model into PSO and proposed Emotional PSO (EPSO) to enhance the search ability of particles and accelerate the convergence speed of PSO. However, the algorithm lacks dynamic adaptability and is prone to getting trapped in local optima [37]. Therefore, this paper proposes an improved PSO algorithm based on EPSO to optimize the regularization parameter and kernel width of the LSSVM. The improved PSO algorithm is expected to overcome the limitations of EPSO and enhance the performance of PSO in solving optimization problems.

Assuming that each particle possesses emotional states and perception abilities, the Weber–Fechner law [38] has been utilized to enhance the performance of PSO. Specifically, particles utilize their emotional states to determine their next actions, and they can perceive stimuli by evaluating the difference between their current position and the historical best position. When the stimulus exceeds the perception threshold, the particle’s emotional state changes, resulting in a stronger response to the stimulus. Three emotional states have been defined for the particles, namely happy, normal, and sad, which correspond to different particle reactions. The emotional state of each particle is updated in each iteration. If the particle’s fitness is higher than that of the previous iteration, its emotional state increases; otherwise, its emotional state decreases. The emotional state of the *i*-th particle in the *t*-th iteration is represented by $eX_{i}^{t}$, and its initial emotional state is a random number. The formula for $eX_{i}^{0}$ can be expressed as:

(19) $$eX_{i}^{0}=rand[-0.1,0.1]$$

The emotional state of the particle is adjusted according to its fitness. If the particle’s fitness is better than the previous iteration, its emotional state increases; otherwise, its emotional state decreases. The increase and decrease of emotional state can be represented as:

(20) $$\Delta^{+}=\frac{\left(f(X_{i}^{t-1})-f(X_{i}^{t}))\cdot (f(X_{i}^{t})-f(gb^{t})\right)}{{\left(f(g\omega^{t})-f(gb^{t})\right)}^{2}}$$

(21) $$\Delta^{-}=\frac{\left(f(X_{i}^{t})-f(X_{i}^{t-1}))\cdot (f(g\omega^{t})-f(X_{i}^{t})\right)}{{\left(f(g\omega^{t})-f(gb^{t})\right)}^{2}}$$

where $X_{i}^{t}$ represents the position of the *i*-th particle during the *t*-th iteration, $gb^{t}$ represents the global best position at the *t*-th iteration, and $g\omega^{t}$ represents the global worst position at the *t*-th iteration.

**Figure 2:**
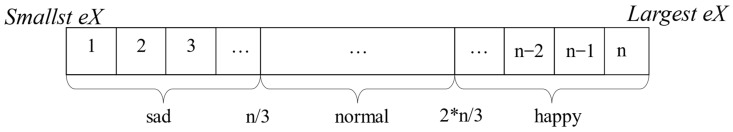
Particle swarm emotional state *eX* sorting.

Regarding particle swarm $pX=\{pX_{i};i=1,2,\cdots ,n\}$, its emotional state is sorted, and all particles are divided into three different emotional states based on the average value of their emotional state, as shown in Figure 2.

Subsequently, the particle’s behavior is determined based on its emotional state. The Weber–Fechner law is employed to describe the particle’s perception ability, which can be expressed as:

(22) $$r_{g}=-k\ln\frac{S(f(gBest)-f(pX_{i}))}{S_{0}}$$

(23) $$r_{h}=-k\ln\frac{S(f(pBest_{i})-f(pX_{i}))}{S_{0}}$$

where $r_{g}$ represents global perception, $r_{h}$ represents historical perception, *k* is a constant factor, *S*(·) is the stimulus function, $S_{0}$ is the stimulus threshold, *gBest* is the historical best position of the particle swarm, and $pBest_{i}$ is the historical position of the *i*-th particle. According to the paper [35], the velocity and position update formulas for a particle in a normal emotional state are:

(24) $$V_{i}^{t+1}=\xi \cdot V_{i}^{t}+c_{1}\cdot r_{1}\cdot (pBest_{i}^{t}-pX_{i}^{t})+c_{2}\cdot r_{2}\cdot (gBest^{t}-pX_{i}^{t})$$

(25) $$pX_{i}^{t}=pX_{i}^{t-1}+V_{i}^{t}$$

When the particle is in the happy state, it will be more energetic in the current position. The update formula of the particle speed and position in the happy state is:

(26) $$V_{i}^{t+1}=\xi \cdot V_{i}^{t}+c_{1}\cdot r_{1}\cdot r_{g}\cdot (pBest_{i}^{t}-X_{i}^{t})+c_{2}\cdot r_{2}\cdot r_{h}\cdot (gBest^{t}-X_{i}^{t})$$

(27) $$pX_{i}^{t}=pX_{i}^{t-1}+V_{i}^{t}$$

When a particle is in a sad emotional state, it primarily focuses on its historical best position and contracts towards it from its current position. However, due to the decreasing fitness over iterations, the particle may become trapped in a local optimum. Based on the psychological model, a particle in a sad emotional state is on the verge of collapse and requires assistance from particles with better emotional states in the swarm to improve its condition. To address this issue, this paper proposes a restart strategy for updating the velocity of sad particles. The restart strategy is as follows:

(28) $$V_{i}^{t}=c_{1}\cdot (u-l)\cdot rand[-1,1]$$

where $c_{1}$ is a constant, *u* represents the upper limit of the particle search range, *l* represents the lower limit of the particle search range, and rand [−1, 1] denotes a random number within the range of [−1, 1]. To maintain both convergence performance and diversity in the particle swarm, a combination of random initialization and the global best position is employed for updating the position of sad particles, which can be expressed as follows:

(29) $$pX_{i}^{t}=\left\{\begin{array}{l} gb^{t-1}\;r<0.5\\ l+(u-l)\cdot rand[-1,1]\;r\geq 0.5 \end{array}\right.$$

**Figure 3:**
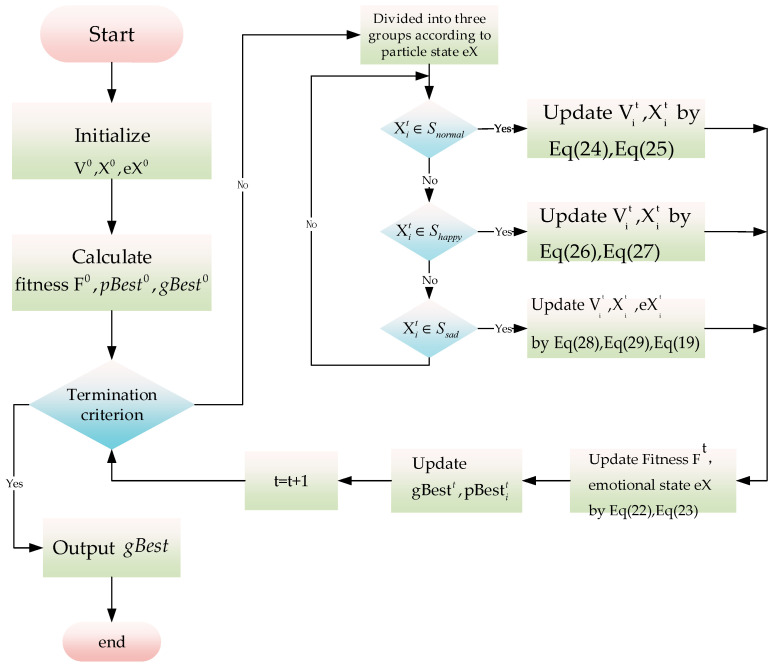
The framework of the IPSO algorithm.

The IPSO algorithm workflow is illustrated in Figure 3.

According to the analysis presented above, the improved PSO algorithm that utilizes the psychological mechanism demonstrates desirable dynamic and convergence performance, as well as enhanced search ability of particles when compared to traditional PSO algorithms. As a result, this study employed the improved PSO algorithm based on the psychological mechanism to optimize the key parameters (γ,σ) of the LSSVM.

### 2.3. Soft Sensor Modeling Based on BDA-IPSO-LSSVM

**Figure 4:**
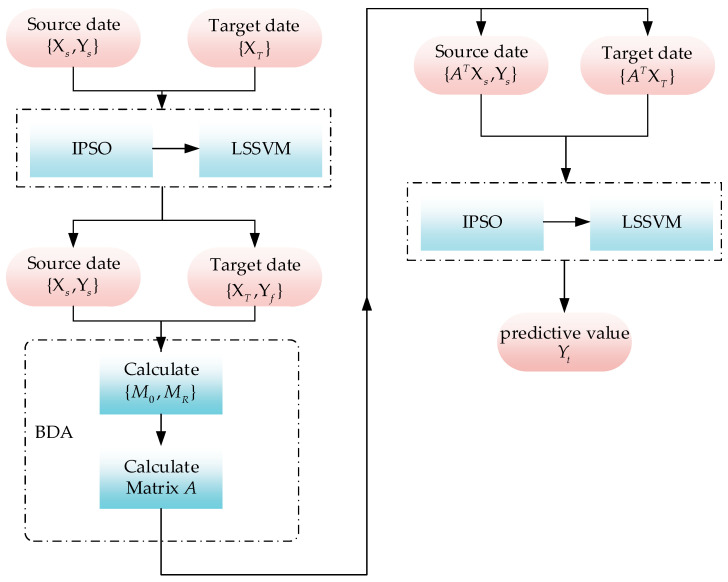
The framework of the BDA-IPSO-LSSVM.

In this study, a soft sensor modeling strategy based on BDA-IPSO-LSSVM is proposed to address the issue of soft sensor model failure caused by the mismatch between training data and actual operating condition data in the fermentation process of *Pichia pastoris*. The proposed strategy utilizes the idea of transfer learning and employs the improved BDA method to match the training data and operating condition data, thereby enhancing the generalization ability and prediction accuracy of the established soft sensor model. Additionally, an improved PSO algorithm is proposed to optimize the established LSSVM-based soft sensor model and overcome the problem of arbitrary local optima in the PSO algorithm. To illustrate the proposed soft sensor modeling strategy based on BDA-IPSO-LSSVM, Figure 4 is provided, which presents a graphical representation of the strategy.

### 2.4. Introduction of the Pichia pastoris Experimental Work

**Figure 5:**
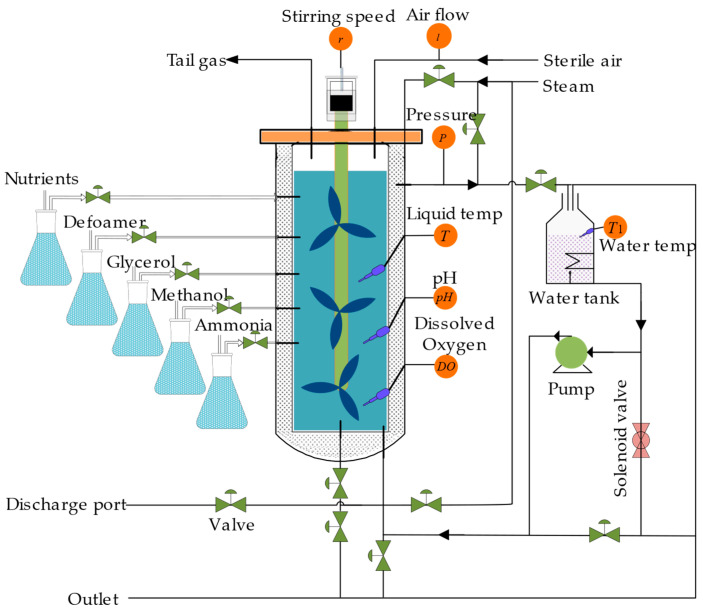
Structure of *Pichia pastoris* fermentation process.

The focus of this study is on *Pichia pastoris*, and *Pichia pastoris* GS115, MutsHis+ strain was selected as the strain. The RTY-C-100L fermenter was used as the fermentation equipment. The input variables for the soft sensor model were chosen based on the analysis of the fermentation process of *Pichia pastoris* using the absolute relation degree method. The stirring speed *v*, temperature *T*, airflow *q*, pH of the fermentation liquid, dissolved oxygen *Do*, and fermenter pressure *p* were selected as input variables, and the concentrations of production *P* and cell concentration *C* were selected as output variables. The fermentation process is shown in Figure 5.

The specific steps for modeling the *Pichia pastoris* fermentation soft sensor model based on BDA method are as follows, where the source domain data and the target domain data are represented by $Q_{s}={\left\{x_{s_{i}},y_{s_{i}}\right\}}_{i=1}^{n}$ and $Q_{t}={\left\{x_{t_{l}},y_{t_{l}}\right\}}_{l=1}^{m}$, respectively:

Step 1: Carry out *Pichia pastoris* fermentation experiments, build the datasets, and normalize the dataset.
Step 2: Establish the IPSO-LSSVM model: the first step is to determine the parameters of the LSSVM model, including regularization parameter γ and kernel width σ. IPSO-LSSVM is an optimization method based on the improved particle swarm algorithm, which automatically selects the optimal model parameters. The IPSO-LSSVM uses the IPSO algorithm proposed in this paper to automatically obtain regularization parameter γ and kernel width σ.
Step 3: Train the IPSO-LSSVM model using labeled source domain data $Q_{s}={\left\{x_{s_{i}},y_{s_{i}}\right\}}_{i=1}^{n}$: labeled source domain data can be used to train the initial IPSO-LSSVM model, which can serve as the starting point for the iterative process, helping to improve the subsequent optimization results.
Step 4: Obtain soft label $y_{f}$ for target domain data $x_{t}$ by iteratively inputting unlabeled data into the IPSO-LSSVM model: Since the target domain data are unlabeled, the unlabeled target domain data $x_{t}$ are input into the IPSO-LSSVM model obtained in step 3 to generate predicted values $y_{t}$, which are then used as soft labels. The soft labels $y_{f}$ are then combined with the target domain data $x_{t}$.
Step 5: Compute the transformation matrix *A* using the source domain data, target domain data, and soft labels as inputs for the improved BDA algorithm: the improved BDA algorithm utilizes the source domain data $Q_{s}$, target domain data $x_{t}$, and target domain data soft labels $y_{f}$ to compute a transformation matrix *A* that matches the source domain data and the target domain data, facilitating the transfer of knowledge from the source domain to the target domain.
Step 6: Input the matched source domain $\left\{A^{T}x_{s},y_{s}\right\}$ and target domain $\left\{A^{T}x_{t}\right\}$ into the IPSO-LSSVM model to obtain the actual predicted key parameters $y_{t}$ in the fermentation process of *Pichia pastoris*.

The specific steps of the *Pichia pastoris* fermentation experiment are as follows:

1. The fermentation system was sterilized and the bacterial strain was cultured according to the requirements of the *Pichia pastoris* fermentation process. The medium was sterilized at 130 °C for 30 min, and the bacterial strain was inoculated by flame when the temperature dropped to 30 °C. The initial fermentation conditions were set: initial tank pressure control at 0.02~0.05 MPa; pH control at 5.0; temperature control at 28 °C; speed set at 300~400 rpm; and airflow velocity control at 150~300 L/M.
2. We selected the stirring speed *v*, temperature *T*, airflow *q*, pH of the fermentation liquid, dissolved oxygen *Do*, and fermenter pressure *P* as auxiliary variables by the absolute relation degree method. All auxiliary variables were transmitted to the database through the distributed control system. The auxiliary variables in this experiment were sampled every 0.5 h.
3. We selected different batches of *Pichia pastoris* fermentation data as data samples. Since the fermentation cycle of *Pichia pastoris* is 90 h, each batch contained 180 data samples. We used auxiliary variables as input variables, cell density and product concentration as output variables, and input the data into the established *Pichia pastoris* fermentation soft sensor model to complete the establishment of the soft sensor model of the *Pichia pastoris* fermentation process and realize real-time prediction of key biological parameters. The root mean square error (*RMSE*), coefficient of determination (*R*^2^), and mean absolute error (*MAE*) and floating point operations (GFLOPs) were used as performance evaluation indicators for the soft sensor model. The calculation formulas are as follows:

(30) $$RMSE=\sqrt{\frac{1}{n}\sum_{i=1}^{n}{\left(y_{pre}^{\left(i\right)}-{\hat{y}}_{real}^{\left(i\right)}\right)}^{2}}$$

(31) $$R^{2}=1-\frac{\sum_{i=1}^{n}{\left(y_{pre}^{\left(i\right)}-y_{real}^{\left(i\right)}\right)}^{2}}{\sum_{i=1}^{n}{\left(y_{real}^{\left(i\right)}-{\hat{y}}_{real}\right)}^{2}}$$

(32) $$MAE=\frac{1}{n}\sum_{i=0}^{n}\left|y_{pre}^{\left(i\right)}-y_{real}^{\left(i\right)}\right|$$

## 3. Result and Discussion

### 3.1. Result

**Figure 6:**
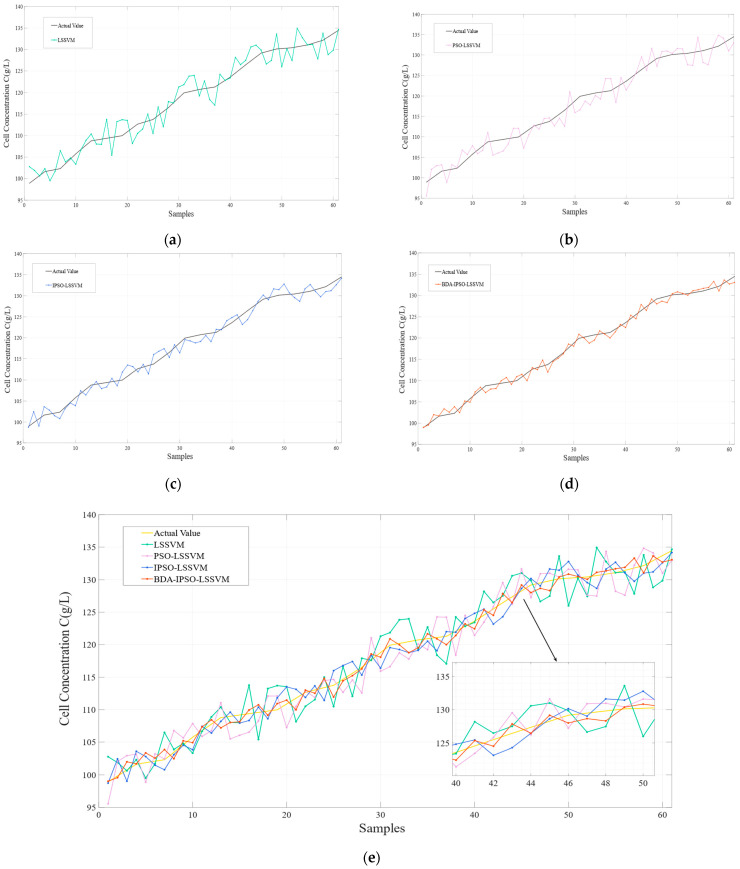
Prediction results of different models for cell concentration: (**a**) LSSVM; (**b**) PSO-LSSVM; (**c**) IPSO-LSSVM; (**d**) BDA-IPSO-LSSVM; (**e**) Combination of the four models.

**Figure 7:**
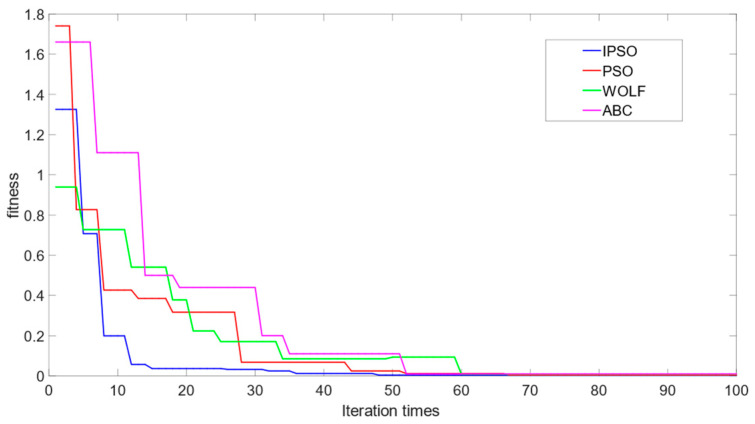
The fitness curve of the IPSO, PSO, WOLF and ABC optimization algorithms.

**Figure 8:**
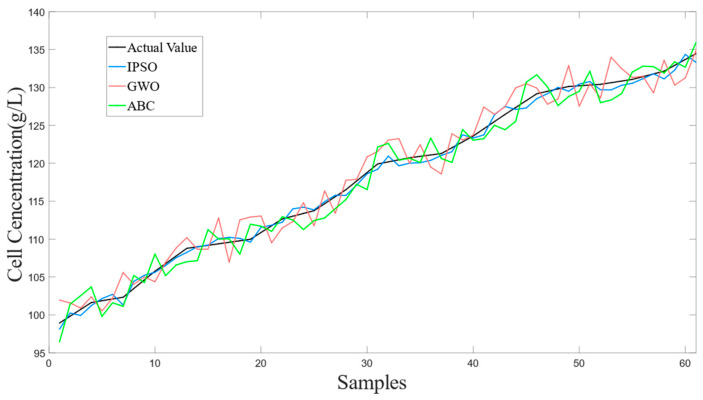
The prediction result of the IPSO-LSSVM, WOLF-LSSVM and ABC-LSSVM.

To better demonstrate the effectiveness of the proposed method in this article, simulations were conducted for the LSSVM soft sensor model, the PSO-LSSVM soft sensor model, the IPSO-LSSVM soft sensor model, and the BDA-IPSO-LSSVM soft sensor model, respectively, to achieve real-time prediction of cell concentration *C* and product concentration *P* of *Pichia pastoris*. The simulation results for cell concentration *C* are shown in Figure 6, where Figure 6a represents the simulation result for LSSVM, using the RBF function as the kernel function, the regularization parameter set to 125, and the RBF kernel width set to 10. PSO and IPSO were introduced to optimize the LSSVM model, with the regularization parameter lower bound set to zero, the upper bound set to 300, the initialization set to 100, the kernel width lower bound set to zero, the upper bound set to 50, the particle swarm size set to 100, and $c_{1}$ set to 0.35, when the number of iterations reaches 200 or the global optimal position is less than 110, it is used as the termination condition of the IPSO algorithm. Finally, the IPSO algorithm was used to obtain the optimized LSSVM regularization parameter of 132.4 and kernel width of 16.4, resulting in the simulation results shown in Figure 6b,c, respectively. The “actual value” in the figures represents the real value measured during the fermentation experiment of *Pichia pastoris*. In order to further illustrate the effectiveness of the proposed IPSO algorithm, Figure 7 shows the fitness curves of the PSO algorithm and the IPSO algorithm. It can be seen that the fitness of the IPSO algorithm is significantly better than that of the PSO algorithm. To further demonstrate the favorable convergence performance and optimization capability of the IPSO algorithm, a comparative analysis was conducted by the IPSO with two classical optimization algorithms, namely Grey Wolf Optimization [39] (GWO) and Artificial Bee Colony [40] (ABC), under identical input conditions. Figure 7 presents the fitness curves of the IPSO, PSO, GWO, and ABC optimization algorithms. It is evident that the IPSO algorithm exhibits a significantly accelerated optimization speed in comparison to the other algorithms. Concerning the prediction results for the cell concentration of *Pichia pastoris*, Figure 8 shows the prediction result of the IPSO-LSSVM, WOLF-LSSVM and ABC-LSSVM. The figure distinctly illustrates that IPSO-LSSVM produces markedly smaller prediction errors compared to the other two algorithms. The results provide substantial evidence that the IPSO algorithm demonstrates pronounced superiority in terms of parameter optimization effectiveness, surpassing both GWO and ABC algorithms.

The improved BDA algorithm was used to further improve the prediction accuracy by adapting the source domain data and target domain data. In the BDA algorithm, the balancing factor μ approaches one as the conditional probability distribution becomes more important and approaches zero as the marginal probability distribution becomes more important. In this simulation, the balancing factor was set to 0.62 to achieve optimal prediction performance. The final simulation result for the BDA-IPSO-LSSVM soft sensor model is shown in Figure 6d. 

By comparing Figure 6a,b, it can be observed that the PSO algorithm can enhance the prediction accuracy of the LSSVM model. However, the prediction effect of the LSSVM model is not ideal and cannot meet the needs of the fermentation industry, significant errors remain present in the model by comparing the predicted value with the actual value. A further comparison of Figure 6b,c indicates that the IPSO algorithm can effectively enhance the prediction accuracy of the model by improving the dynamic performance of the PSO algorithm, and the IPSO algorithm reduces the prediction error of the model. The result presented in Figure 6d demonstrates that the proposed IBDA algorithm is effective in reducing the distribution distance between the source domain and the target domain and makes the predicted value of the model meet the actual fermentation production needs. It can be seen that the proposed BDA-IPSO-LSSVM model has good prediction accuracy in Figure 6e.

**Figure 9:**
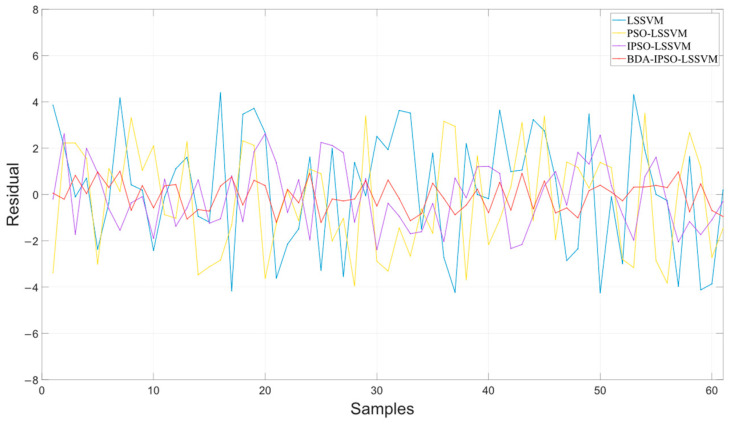
The residuals of the different soft sensor models in predicting the cell concentration of *Pichia pastoris*.

Figure 9 shows the residuals of the different soft sensor models in predicting the cell concentration of *Pichia pastoris*. The prediction performance of the soft sensor models is shown in Table 1. It can be intuitively seen in Figure 8 and Table 1 that the BDA-IPSO-LSSVM residual is relatively small compared to other models, and it performs well in the *RMSE*, *R*^2^, and *MAE* performance metrics. GFLOPs reflects the complexity of the model. From the value of GFLOPs of each model in Table 1, it can be seen that the proposed hybrid model does not increase the complexity of the model.

**Figure 10:**
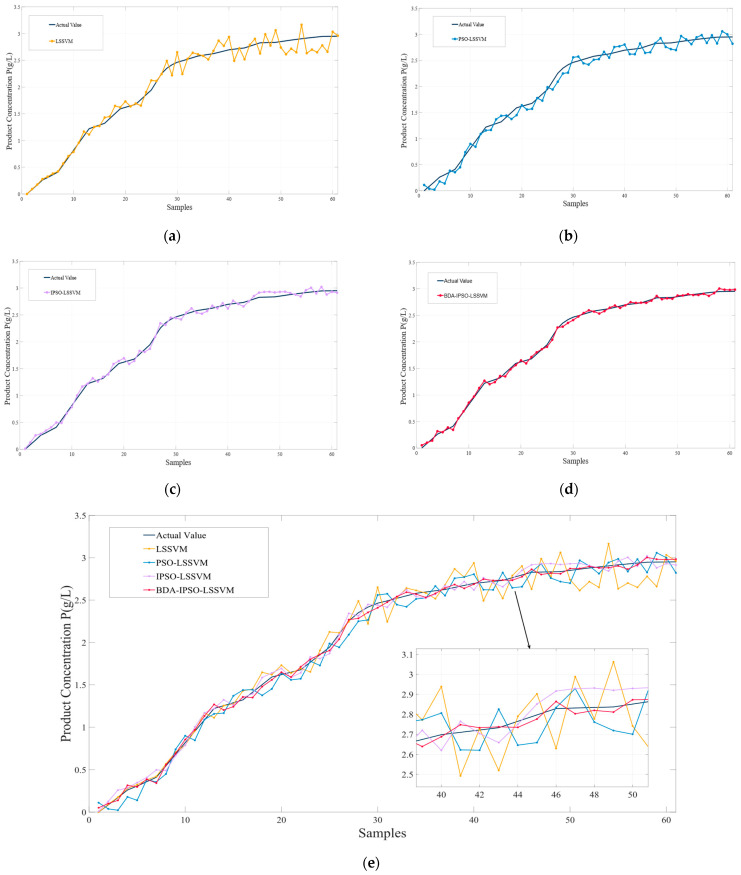
Prediction results of different models for product concentration: (**a**) LSSVM; (**b**) PSO-LSSVM; (**c**) IPSO-LSSVM; (**d**) BDA-IPSO-LSSVM; (**e**) Combination of the four models.

**Figure 11:**
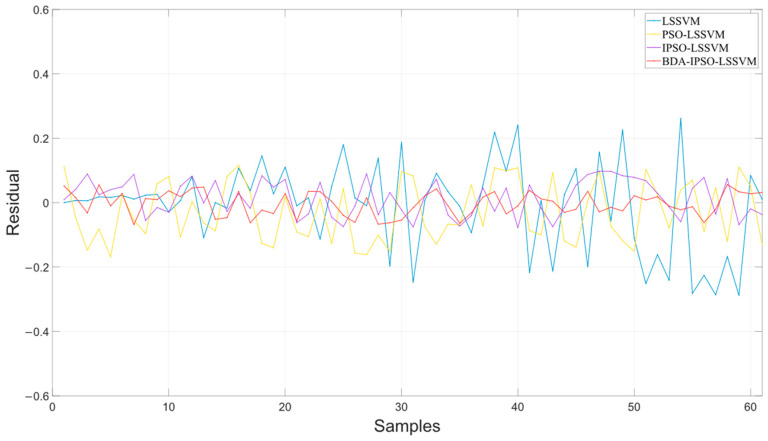
The residuals of the different soft sensor models in predicting the product concentration of *Pichia pastoris*.

To further illustrate the effectiveness of the proposed BDA-IPSO-LSSVM model, similar to Figure 6, Figure 10 illustrates the predicted values of the Pichia pastoris product concentration. Specifically, Figure 10 presents the results of the four different soft sensor models, while Figure 10a–d show the prediction results of the LSSVM model, PSO-LSSVM model, IPSO-LSSVM model, and BDA-IPSO-LSSVM model for product concentration during the fermentation process of *Pichia pastoris*. As evidenced by Figure 10e, the BDA-IPSO-LSSVM model proposed in this paper exhibits strong predictive performance in regard to the key parameters during the fermentation process of *Pichia pastoris*. Figure 11 displays the residuals of the different soft sensor models, and Table 2 presents the performance metrics of the prediction results. By means of comparison, it can be concluded that the proposed BDA-IPSO-LSSVM soft sensor model exhibits superior prediction accuracy compared to the other models.

### 3.2. Discussion

This paper proposes a soft sensor model of *Pichia pastoris* based on LSSVM. Through Figure 6 and Figure 10, it can be seen that the proposed soft sensor model has good predictive performance and can better realize the real-time monitoring of the fermentation process of *Pichia pastoris*. This paper first proposes an IPSO algorithm to optimize the parameters of LLSVM to achieve good prediction performance of LSSVM. Figure 7 and Figure 8 show that compared with the GWO and ABC optimization algorithms, the IPSO algorithm proposed in this paper has good dynamic performance and convergence performance: it better solves the optimization problem of LSSVM parameters and realizes an accurate prediction of the LSSVM model. Second, this paper uses the BDA method in transfer learning to match the source domain data and target domain data, and realizes the accurate prediction of the *Pichia pastoris* soft sensor model under different working conditions. In Figure 9 and Figure 11, it can be seen that, compared with the model without transfer, the proposed hybrid model reduces the prediction error of the model to a large extent and solves the problem of model failure under different working conditions. It can be seen from Table 1 and Table 2 that the hybrid model proposed in this paper has good performance in various evaluation indicators. Moreover, the simulation results show that the hybrid model proposed in this paper has good predictive performance and can realize the real-time and accurate prediction of the cell concentration and product concentration in the fermentation process of *Pichia pastoris*, which greatly improves the production efficiency of *Pichia pastoris* fermentation products.

## 4. Conclusions

This study has proposed a novel soft sensor modeling strategy based on BDA-IPSO-LSSVM to address the issue of data distribution differences resulting from different operating conditions during the fermentation process of *Pichia pastoris*. The proposed strategy employs the BDA method and a fuzzy set-based improvement to reduce the distribution differences and improve the generalization ability of the traditional soft sensor model. Additionally, an improved PSO algorithm is proposed to optimize the established LSSVM-based soft sensor model, which addresses the issue of PSO algorithms becoming trapped in local optima and results in a significant improvement in the prediction accuracy of the soft sensor model. The experimental results demonstrate that the proposed BDA-IPSO-LSSVM soft sensor model exhibits strong performance in terms of the *RMSE*, *R*^2^, and *MAE* prediction performance indicators. The soft sensor model can effectively predict the key parameters of *Pichia pastoris* fermentation in real-time, including cell concentration and product concentration. The proposed strategy offers a promising solution to the issue of soft sensor model failure caused by the mismatch between training data and actual operating condition data and has potential applications in the fermentation industry. Future studies may explore the generalization of the proposed strategy to other fermentation processes or even other fields. The main limitation at present is that only one source domain and one target domain can be adapted, and the IPSO can be optimized for a single objective. In the future, we aim to research the transfer learning from the data of multiple historical operating conditions to further reduce the difference between data, and generalization of IPSO to multiobjective optimization problems.

## Figures and Tables

**Table 1:** Prediction performance indexes of different soft sensor models in predicting cell concentration.

	*RMSE*	*R* ^2^	*MAE*	GFLOPs
LSSVM	2.3356	0.9425	2.1929	0.0014
PSO-LSSVM	2.1830	0.9572	1.9425	0.0016
IPSO-LSSVM	1.5902	0.9779	1.4154	0.0016
BDA-IPSO-LSSVM	1.0485	0.9912	0.8554	0.0016

**Table 2:** Prediction performance indexes of different soft sensor models in predicting product concentration.

	*RMSE*	*R* ^2^	*MAE*	FLOPs
LSSVM	0.1397	0.9773	0.1046	0.0013
PSO-LSSVM	0.0973	0.9890	0.0887	0.0015
IPSO-LSSVM	0.0569	0.9962	0.0508	0.0015
BDA-IPSO-LSSVM	0.0368	0.9984	0.0322	0.0015

## Data Availability

The dataset in this article is unavailable because it involves privacy.

## Abbreviations

BDA	balanced distribution adaptation
LSSVM	least squares support vector machine
IPSO	improved particle swarm optimization
LSTM	Long Short Term Memory
GWO	Grey Wolf Optimization
ABC	Artificial Bee Colony
MMD	Maximum Mean Discrepancy
H	Reproducing Kernel Hilbert Space
KKT	Karush–Kuhn–Tucker
PSO	Particle Swarm Optimization
EPSO	Emotional Particle Swarm Optimization
*RMSE*	Root Mean Square Error
*R* ^2^	R-Square
FLOPs	Floating Point Operations
*MAE*	Mean Absolute Error

## Nomenclature

$Q_{s}$	the source domain data
$x_{s_{i}}$	*i*-th input source domain data
$y_{s_{i}}$	*i*-th output source domain data
*n*	the amount of data in the source domain
$Q_{t}$	target domain data
$x_{t_{j}}$	*j*-th input source domain data
$y_{t_{j}}$	*j*-th output source domain data
*m*	the amount of data in the target domain
$\chi_{s}$	the source domain future space
$\chi_{t}$	the target domain future space
$\gamma_{s}$	the source domain label space
$\gamma_{t}$	the target domain label space
$P_{s}(x_{s})$	the source domain marginal distribution
$P_{t}(x_{t})$	the target domain marginal distribution
$P_{s}(y_{s}|x_{s})$	the source domain conditional distribution
$P_{t}(y_{t}|x_{t})$	the target domain conditional distribution
μ	the balance factor
$Q_{s}^{c}$	the sample set of class c in the source domain
$Q_{t}^{c}$	the sample set of class c in the target domain
*C*	the number of classes
*c*	the *c*-th classes
$n_{c}$	the number of samples in $Q_{s}{}^{c}$
$m_{c}$	the number of samples in $Q_{t}{}^{c}$
$y_{i}^{z}$	the *z*-th source domain i-th data
$P_{5}^{z}$	the *z*-th source domain 5-th percentile values
$P_{50}^{z}$	the *z*-th source domain 50-th percentile values
$P_{95}^{z}$	the *z*-th source domain 95-th percentile values
$\alpha_{ip}^{z}$	the membership degree of $y_{i}^{z}$ in class p in the source domain
$\alpha_{iq}^{t}$	the membership degree of $y_{i}^{t}$ in class q in the target domain
$M_{c}$	the matrices for maximum mean discrepancy
λ	the regularization parameter
*A*	the transformation matrix
*X*	the input matrix composed of $x_{s_{i}}$ and $x_{t_{j}}$
$||•|{|}_{F}^{2}$	the Hilbert–Schmidt norm
*E*	the identity matrix
ϕ	the Lagrange multiplier
ω	the weight vector
φ(i)	a nonlinear function that maps the data to a high-dimensional space
γ	the regularization parameter
$e_{i}$	the error introduced by the samples
*b*	the constant bias
L(ω,b,e,α)	the LSSVM optimization objective
$\alpha_{i}$	the Lagrange multiplier for the *i*-th constraint
$I_{l}$	an *l∗l* identity matrix
Ω	the kernel matrix
σ	the kernel width
$K(x_{i},x_{j})$	the RBF kernel
$eX_{i}^{t}$	The emotional state of the *i*-th particle in the *t*-th iteration
$\Delta^{+}$	the increase of emotional state
$\Delta^{-}$	the increase of emotional state
$X_{i}^{t}$	the position of the *i*-th particle during the *t*-th iteration
$gb^{t}$	the global best position at the *t*-th iteration
$g\omega^{t}$	the lobal worst position at the *t*-th iteration
pX	the particle swarm
$pX_{i}$	the i-th particle position
$r_{g}$	the global perception
$r_{h}$	The historical perception
*k*	a constant factor
*S*(·)	the stimulus function
$S_{0}$	the stimulus threshold
*gBest*	the historical best position of the particle swarm
$pBest_{i}$	the historical position of the *i*-th particle
μ	the upper limit of the particle search range
*l*	the lower limit of the particle search range
*v*	The stirring speed
*T*	temperature
*q*	airflow
*Do*	dissolved oxygen
*C*	cell concentration
*p*	fermenter pressure
*P*	the concentrations of production
$y_{pre}^{\left(i\right)}$	the model *i*-th prediction data
$y_{real}^{\left(i\right)}$	*i*-th real data
